# Virulence, antimicrobial and heavy metal tolerance, and genetic diversity of *Vibrio cholerae* recovered from commonly consumed freshwater fish

**DOI:** 10.1007/s11356-019-05287-8

**Published:** 2019-07-19

**Authors:** Mengjie Xu, Jinrong Wu, Lanming Chen

**Affiliations:** 1 Key Laboratory of Quality and Safety Risk Assessment for Aquatic Products on Storage and Preservation (Shanghai), China Ministry of Agriculture, College of Food Science and Technology, Shanghai Ocean University, 999 Hu Cheng Huan Road, Shanghai, 201306 People’s Republic of China; 2 College of Life Science and Technology, Xinjiang University, Xinjiang, 830000 People’s Republic of China

**Keywords:** *Vibrio cholerae*, Virulence, Antimicrobial susceptibility, Heavy metal tolerance, Genotyping, Freshwater fish

## Abstract

*Vibrio cholerae* is a leading waterborne pathogen worldwide. Continuous monitoring of *V. cholerae* contamination in aquatic products and identification of risk factors are crucial for assuring food safety. In this study, we determined the virulence, antimicrobial susceptibility, heavy metal tolerance, and genetic diversity of 400 *V. cholerae* isolates recovered from commonly consumed freshwater fish (*Aristichthys nobilis*, *Carassius auratus*, *Ctenopharyngodon idellus*, and *Parabramis pekinensis*) collected in July and August of 2017 in Shanghai, China. *V. cholerae* has not been previously detected in the half of these fish species. The results revealed an extremely low occurrence of pathogenic *V. cholerae* carrying the major virulence genes *ctxAB* (0.0%), *tcpA* (0.0%), *ace* (0.0%), and *zot* (0.0%). However, high incidence of virulence-associated genes was observed, including the RTX toxin gene cluster (*rtxA*-*D*) (83.0–97.0%), *hlyA* (87.8%), *hapA* (95.0%), and *tlh* (76.0%). Meanwhile, high percentages of resistance to antimicrobial agents streptomycin (65.3%), ampicillin (44.5%), and rifampicin (24.0%) were observed. Approximately 30.5% of the isolates displayed multidrug resistant (MDR) phenotypes with 42 resistance profiles, which were significantly different among the four fish species (MARI, *P* = 0.001). Additionally, tolerance of isolates to heavy metals Hg^2+^ (49.3%), Zn^2+^ (30.3%), and Pb^2+^ (12.0%) was observed. The enterobacterial repetitive intergenic consensus-polymerase chain reaction (ERIC-PCR)-based fingerprinting of the 400 *V. cholerae* isolates revealed 328 ERIC-genotypes, which demonstrated a large degree of genomic variation among the isolates. Overall, the results of this study support the need for food safety risk assessment of aquatic products.

**Electronic supplementary material:**

The online version of this article (10.1007/s11356-019-05287-8) contains supplementary material, which is available to authorized users.

## Introduction

*Vibrio cholerae* can cause cholera, a severe diarrheal disease that can be quickly fatal if untreated and is typically transmitted via contaminated water and person-to-person contact (Baker-Austin et al. 2018). It was estimated that *V. cholera* caused roughly 2.9 million cases of cholera and 95,000 deaths annually worldwide between 2008 and 2012 (Ali et al. 2015). The bacterium is found growing in aquatic environment or aquatic products such as crustaceans and fish (Vezzulli et al. 2010). Previous studies highlighted the link between cholera outbreaks and the consumption of raw, undercooked, or mishandled fish products contaminated by *V. cholerae*. For instance, consumption of dried fish was significantly associated with the 1997 cholera epidemic in a rural area (Ifakara) in southern Tanzania (Acosta et al. 2001). It has also been reported that the fresh fish imported from Nigeria contributed to the domestic cholera in Germany in 2001 (Schurmann et al. 2002). Since 2002, China has become the largest producer and exporter in the world for fishery products, which accounted for 62.6% (69,012,500 tons) of the global amount in 2016. Freshwater aquaculture is an important component of Chinese fishery production, and accounted for 48.4% of the total fishery output value in China (Zhang et al. 2018). *C*. *idellus* (known as grass carp) is the most important freshwater cultured fish with the largest production in China, and its total output exceeded 5,676,235 tons in 2015 (Jia et al. 2016). *C*. *auratus* (known as crucian carp) has been cultured in China for several hundred years (Jing et al. 2016), and recently played an important role in the international fish trade with a total output of 2,912,258 tons in 2015 (Jia et al. 2016). *A*. *nobilis* (known as bighead carp) and *P*. *pekinensis* (known as white bream) are also two of the most dominant fish species in freshwater aquaculture in China. Therefore, continuous monitoring of *V. cholerae* contamination in freshwater fish is imperative for food safety control.

Two major virulence genes that encode cholerae toxin (CT) and toxin coregulated pilus (TCP) have been identified in epidemic *V. cholerae* strains of serotypes O1 and O139. These toxin genes are carried by a lysogenic filamentous bacteriophage (CTX prophage) that can integrate into *V. cholerae* chromosomes (Waldor and Mekalanos 1996). The non-epidemic *V. cholerae* strains, referred to non-O1/O139, can cause sporadic episodes of diarrhea and gastrointestinal infection (Austin 2010; Ceccarelli et al. 2015). The genes encoding virulence-associated factors that contribute to the pathogenicity of *V. cholerae* include the zonula occludens toxin (*zot*) (Preeprem et al. 2014), accessory cholera enterotoxin (*ace*) (Briquaire et al. 2017), RTX toxin gene cluster (*rtxA*-*D*) (Lin et al. 1999), El Tor hemolysin (*hlyA*) (Ruenchit et al. 2017), thermolabile hemolysin (*tlh*) (Fiore et al. 1997), hemagglutinin protease (*hapA*) (Halpern et al. 2003), and two morphologically distinct types of pili, namely, mannose-sensitive hemagglutination (MSHA) pili (*msha*) (Moorthy and Watnick 2004) and putative type IV pilus (*pil*) (Fullner and Mekalanos 1999).

Antimicrobial agent treatment can effectively control outbreaks and prevalence of infectious diseases caused by pathogenic microorganisms. However, the inappropriate usage of antimicrobial drugs in aquaculture contributed to the development of antimicrobial-resistant bacteria and imposed potential threat upon human health due to the dissemination of antimicrobial resistance (Woolhouse and Farrar 2014). The aquatic environment is a reservoir of *V. cholerae* and might be an important source of resistant strains (Baron et al. 2017). Numerous previous studies have been focused on the detection of *V. cholerae* from various aquatic environments. For instance, Sulca et al. reported that two *V. cholera* isolates from Lima (Peru) seawater were resistant to 12 antimicrobial drugs, including ampicillin (AMP), penicillin, amoxicillin, nitrofurantion, kanamycin (KAN), amikacin, aztreonam, ciprofloxacin, gentamicin (CN), co-trimoxazole, ceftazidime, and nalidixic acid (Sulca et al. 2018). Bhuyan et al. also reported that 107 *V. cholerae* strains originated from different aquatic environment (including river water, canal water, pond water, and hand-pump water) in India showed varying degrees of resistance to AMP, co-trimoxazole, nalidixic acid, polymyxin-B, streptomycin (STR), ciprofloxacin, and tetracycline (TET) (Bhuyan et al. 2016). Antimicrobial-resistant *V. cholerae* isolated from aquacultured animals such as shrimps and shellfish have also been reported. For example, He et al. analyzed 42 *V. cholerae* isolates recovered from shrimp collected in 2013 and 2014 in Shanghai, China, and found that 33.3%, 21.4%, 19.1%, 9.5%, and 9.5% of the isolates were resistant to rifampicin (RIF), STR, KAN, AMP, and TET, respectively. Moreover, 25 isolates (59.5%) had MDR phenotypes (He et al. 2015).

*V. cholerae* has been isolated from approximately 30 fish species belonging to nine different orders within the *Actinopterygii* class (Halpern and Izhaki 2017). The bacterium was found to reside in healthy fish intestines (Halpern and Izhaki 2017). For instance, *V. cholerae* was detected from the intestines of ten freshwater fish species collected in Israel in 2008, including *Astatotilapia flaviijosephi*, *Barbus longiceps*, *Carasobarbus canis*, *C*. *idella*, *Cyprinus carpio*, *Mugil cephalus*, *Myripristis murdjan*, *Oreochromis aureus*, *Sarotherodon galilaeus*, and *Tilapia* sp. and *Tilapia zilli* (Senderovich et al. 2010). Recently, *V. cholerae* was also isolated from four freshwater fish species (*C*. *auratus*, *C*. *idella*, *Cyprinus carpio*, and *Hypophthalmichthys molitrix*) collected in Chengdu, China (Li et al. 2017). Laviad-Shitrit et al. reported that 95.8% of *V. cholerae* isolates (non-O1/O139) (*n* = 48) derived from fish intestines showed high minimal inhibitory concentration (MIC) (MIC_90_ of 16 μg/mL) to doxycycline (Laviad-Shitrit et al. 2018).

Water contaminated with heavy metals may enhance selection for antibiotic resistance and vice versa (Baker-Austin et al. 2006; Matyar 2012). The commonly detected heavy metals in the environment include chromium (Cr), zinc (Zn), cadmium (Cd), copper (Cu), mercury (Hg), nickel (Ni), and lead (Pb) (Wuana and Okieimen 2011). Nevertheless, very little information is available concerning the tolerance of *V. cholerae* to heavy metals to date. In our prior study, our data revealed that *V. cholerae* isolates Chn64, Chn86, Chn91, Chn92, and Chn108 derived from the surface water of Yangtze River Estuary in Shanghai, China, showed high levels of tolerance to Hg, Cd, and Cu; Hg, Cd, and Cu; Hg, Cd, Pb, and Cu; and Hg, Cd, Zn, Pb, and Cu; as well as Hg, Cd, and Pb, respectively. Meanwhile, the isolates Chn64, Chn91, Chn92, and Chn108 were also resistant to AMP; AMP and RIF; and AMP and RIF; as well as AMP, sulfamethoxazole, and STR, respectively (Song et al. 2013). We hypothesized that there could be high incidence of antimicrobial and/or heavy metal–resistant *V. cholerae* in freshwater fish.

In this study, we investigated the virulence, antibiotic, and heavy metal tolerance of 400 *V. cholerae* strains isolated from four commonly consumed freshwater fish (*A*. *nobilis*, *C*. *auratus*, *C*. *idellus*, and *P*. *pekinensis*) sampled in July and August of 2017 in Shanghai, China. Additionally, we also obtained and compared fingerprinting profiles of the 400 *V. cholerae* isolates using the ERIC-PCR assay to address their phylogenetic relatedness for better understanding of genome evolution of the bacterium.

## Materials and methods

### Sample collection

The four commonly consumed freshwater fish were sampled in July and August of 2017 from two largest fish markets located in Shanghai, China, including the Jiayan Aquatic Market (31° 19′ 57.61″ N, 121° 10′ 53.05″ E) and Jiangyang Aquatic Market (31° 21′ 25.90″ N, 121° 26′ 50.68″ E). Forty fish samples comprising of *A*. *nobilis* (*n* = 10), *C*. *auratus* (*n* = 10), *C*. *idellus* (*n* = 10), and *P*. *pekinensis* (*n* = 10) were collected into sterile sealed bags (Nanjing Maojie Microbial Technology Co., Ltd., Nanjing, China), and immediately transported in ice boxes (700 × 440 × 390 mm) to the laboratory in Shanghai Ocean University, Shanghai, China, for analysis.

### Isolation of *V. cholerae*

*V. cholerae* was isolated and identified in accordance with the instructions of the Chinese Government Standard (SN/T 1022-2010) and the Bacteriological Analytical Manual of the US Food and Drug Administration (8th Edition, Revision A, 1998) as described previously (Song et al. 2013). Briefly, 25 g of each fish intestine sample was rinsed with 225 mL sterile 1× phosphate buffer saline (PBS, pH 7.4–7.6, Shanghai Sangon Biological Engineering Technology and Services Co., Ltd., Shanghai, China), and then homogenized for 2 min using a stomacher (Bagmixer 400 W, Interscience, Saint Nom la Bretèche, France). Serial tenfold dilutions were prepared up to 1:10^5^ dilution, and 100 μL of each dilution was spread on thiosulfate citrate bile salts sucrose (TCBS; Beijing Land Bridge Technology Co., Ltd., Beijing, China) agar plates, which were incubated at 37 °C for 24 h. Yellow, flat, and shiny colonies that were 2 to 3 mm in diameter on the TCBS agar plates were picked out for further analysis.

### Identification of *V. cholerae*

*V. cholerae* isolates were also identified by biochemical tests, including the arginine dihydrolase test and the esculin hydrolysis test (Choopun et al., 2002; Thornley 1960) using the double-arginine hydrolase medium (pH 6.8, 3.0% NaCl) and the esculin medium (pH 7.3, 3.0% NaCl) (Muwei Biotechnology Co., Ltd., Shanghai, China), respectively. After inoculation, the former medium was covered with sterile mineral oil (Shanghai Sangon Biological Engineering Technology and Services Co., Ltd., Shanghai, China), and then incubated at 37 °C for 24 h. Appearance of a red color was considered as a positive reaction, while the blackening of the latter medium indicated a positive reaction. *V. cholerae* isolates that were detected negative in the two tests, showing deep yellow and brown, respectively, were picked out for the further identification by the PCR assay and DNA sequencing analysis.

The oligonucleotide primers (VHMF and VHA-AS5, Table 1) targeting the *V. cholerae*-specific gene *lolB* were synthesized by Shanghai Sangon Biological Engineering Technology and Services Co., Ltd. (Shanghai, China). The *lolB* gene with an expected amplicon size of 516 bp was amplified according to the method described previously (Lalitha et al. 2008) with slight modification. Briefly, PCR reaction mixture contained 8 μL of DNase/RNase-free deionized water (Tiangen Biotech Co., Ltd., Beijing, China), 10 μL of 2× Taq Master Mix (Novoprotein Technology Co., Ltd., Shanghai, China), 0.5 μL of each primer (VHMF and VHA-AS5), and 1 μL of DNA template. PCR was carried out under the following conditions: initial denaturation at 94 °C for 5 min, followed by 30 cycles consisting of denaturation at 94 °C for 1 min, annealing at 57 °C for 1 min, and extension at 72 °C for 1 min. All PCR reactions were performed in a Mastercycler® pro PCR thermal cycler (Eppendorf, Hamburg, Germany). Amplicons were analyzed by electrophoresis with a 2.0% agarose gel, and visualized and recorded using a UVP EC3 Imaging system (UVP LLC, Upland, CA, USA). *V. cholerae* GIM 1.449 (*lolB*^+^) (Guangdong Culture Collection Center, Guangzhou, China) was used as a positive control strain.

**Table 1:** Oligonucleotides used in this study

Primer	Sequence (5′→3′)	Amplicon size (bp)	Reference
VHMF	TGGGAGCAGCGTCCATTGTG	516	Lalitha et al. (2008)
VHA-AS5	CAATCACACCAAGTCACTC		
27F	GAGAGTTTGATCCTGGCTCAG	~ 1540	Weisburg et al. (1991)
1492R	TACGGCTACCTTGTTACGAC		
*ctxAB*-F	TGAAATAAAGCAGTCAGGTG	778	McGrath et al. (2006)
*ctxAB*-R	GGTATTCTGCACACAAATCAG		
*tcpA*-F	ATGCAATTATTAAAACAGCTTTTTAAG	675	Kumar et al. (2010)
*tcpA*-R	TTAGCTGTTACCAAATGCAACAG		
*ace*-F	TAAGGATGTGCTTATGATGGACACCC	316	Singh et al. (2002)
*ace*-R	CGTGATGAATAAAGATACTCATAGG		
*zot*-F	TCGCTTAACGATGGCGCGTTTT	947	Tulatorn et al. (2018)
*zot*-R	AACCCCGTTTCACTTCTACCCA		
*rtxA*-F	GGGATACAATGCCCTCTGGCA	977	Rivera et al. (2001)
*rtxA*-R	TGGGTTGGCGGTTGGATTTTAC		
*rtxB*-F	ATTCATTTTTATTTAAGTGTCATCA	400	This study
*rtxB*-R	TTTCGCTCAGCACTCTTT		
*rtxC*-F	ATGTCTATTACACATCAACCTGCAA	437	This study
*rtxC*-R	CGGATACAGCGGTCATTT		
*rtxD*-F	ATCATGAAGCGTTTCTTTGGTCAAA	334	This study
*rtxD*-R	CGCCCAAGGTATCAAGAGTCAG		
*tlh*-F	TGGGAGTGGGCAAAGAAT	274	This study
*tlh*-R	AAAGGCTATCGCCAAACG		
*hlyA*-F	CCAAGTGGTGAAGCGGCGGAC	393	Kumar et al. (2010)
*hlyA*-R	TTCGCTGTTTGCCGGTGCCG		
*hapA*-F	CGTTAGTGCCCATGAGGTC	207	This study
*hapA*-R	CGTGACGGCTGATCGAAAT		
*pilA*-F	GCGATTGCAATTCCTCAA	227	This study
*pilA*-R	CCTAATGCACCTGATGCT		
*mshA*-F	CGCTAGATACTTCGAGTGAG	189	This study
*mshA*-R	TACCACAAGCAGTTCCAG		
ERIC1R	ATGTAAGCTCCTGGGGATTCAC		Rivera et al. (1995)
ERIC2	AAGTAAGTGACTGGGGTGAGCG		

Bacterial 16S ribosomal RNA (rRNA) gene was also amplified using universal bacterial primers 27F and 1492R (Weisburg et al. 1991). A 25-μL reaction mixture contained 12.5 μL of 2× Taq Master Mix (Novoprotein Technology Co., Ltd., Shanghai, China), 1.25 μL of each primer (27F and 1492R), and 1 μL of DNA template. The thermal cycler was used at the following setting: initial denaturing at 94 °C for 3 min, followed by 30 cycles of denaturing at 94 °C for 40 s, annealing at 55 °C for 45 s, elongation at 72 °C for 2 min. The PCR products were analyzed as described above.

The PCR products were purified and sequenced by Shanghai Sangon Biological Engineering Technology and Services Co., Ltd. (Shanghai, China). DNA sequences were analyzed using the Basic Local Alignment Search Tool (BLAST) of the National Center for Biotechnology Information (NCBI) (available online: https://www.ncbi.nlm.nih.gov/).

### Genomic DNA extraction

*V. cholerae* isolates were cultured in Luria-Bertani (LB) broth (Beijing Land Bridge Technology Co., Ltd., Beijing, China) (pH 8.5, 3.0% NaCl) overnight at 37 °C. Bacterial lysate was prepared according to the method described previously (Letchumanan et al. 2015). Briefly, 5 μL of bacterial culture was heated in 50 μL of ultrapure water at 95 °C for 10 min, and then transferred on ice for cooling. After centrifugation at 12,000 rpm for 5 min, the resulting supernatant was used as DNA template for PCR assays.

### Detection of virulence and virulence-associated genes

The major virulence genes (*ctxAB* and *tcpA*) (Kumar et al. 2010; McGrath et al. 2006) and virulence-associated genes (*ace*, *zot*, *rtxABCD*, *hapA*, *hlyA*, *tlh*, *mshA*, and *pilA*) (Singh et al. 2002; Tulatorn et al. 2018) were detected by the PCR assay. PCR reactions were performed as described above, but with different annealing temperatures and elongation times based on melting temperatures of primer pairs and predicted sizes of PCR products. The primers were synthesized as described above. *V. cholerae* ATCC 14035 (*ctxAB*^+^, *tcpA*^+^, *ace*^+^, and *zot*^+^) and GIM 1.449 (*ctxAB*^−^, *rtxA*^+^, *rtxB*^+^, *rtxC*^+^, *rtxD*^+^, *hlyA*^+^, *tlh*^+^, and *hapA*^+^), which were isolated from clinical and environmental cases, respectively, were used as positive control strains as described previously (Boustanshenas et al. 2013). PCR products were confirmed by DNA sequencing at Shanghai Sangon Biological Engineering Technology and Services Co., Ltd. (Shanghai, China). All the oligonucleotide primers used in this study are listed in Table 1.

### Antibiotic susceptibility and heavy metal tolerance assays

*V. cholerae* isolates were measured for in vitro susceptibility to ten antimicrobial agents (Oxoid, UK) according to the method described previously (Hu and Chen 2016; Tang et al. 2014). Ten antimicrobial agents were AMP, CHL, KAN, RIF, SPT, STR, TET, TM, and sulfamethoxazole plus trimethoprim (SXT). Tolerance of *V. cholerae* isolates to eight heavy metals NiCl_2_, CrCl_3_, CdCl_2_, PbCl_2_, CuCl_2_, ZnCl_2_, MnCl_2_, and HgCl_2_ (Analytical Reagent, Sinopharm Chemical Reagent Co., Ltd, Shanghai, China) was also determined according to the method described previously (Hu and Chen 2016). *Escherichia coli* strains ATCC25922 and K12 (Institute of Industrial Microbiology, Shanghai, China) were used as quality control strains in antibiotic and heavy metal resistance tests, respectively (Matyar 2012; Song et al. 2013).

### ERIC-PCR assay

Strain taxonomy was determined by ERIC-PCR with the primer set ERIC1R and ERIC2 (Rivera et al. 1995) (Table 1). A 20-μL reaction mixture contained 10 μL of 2× Taq Master Mix (Novoprotein Technology Co., Ltd., Shanghai, China), 1 μL of each primer, and 2 μL of DNA template. The ERIC-PCR was performed under the following conditions: denaturation at 95 °C for 30 s, annealing at 52 °C for 1 min, and extension at 65 °C for 8 min. Following 32 reaction cycles, reaction mixtures were further incubated at 65 °C for an additional 16 min. Six microliters of each amplicon was electrophoresed at 100 V for about 45 min on a 1.0% agarose gel. Amplified DNA fragments were visualized and recorded as described above.

### Statistical analysis

Data analysis was performed using the SPSS statistical analysis software version 17.0 (SPSS Inc., Chicago, USA). The multiple antimicrobial resistance index (MARI) of an isolate is defined as *a*/*b*, where *a* represents the number of antibiotics to which the isolate was resistant, and *b* represents the number of antibiotics to which the isolate was subjected (Krumperman 1983). One-way analysis of variance (ANOVA) followed by appropriate post-hoc test (Tukey) was performed to determine significant differences between the four different fish samples and MARI of resistant isolates, and *P* < 0.05 was considered statistically significant. DNA banding patterns generated by the ERIC-PCR were analyzed using the BioNumerics 7.6 software (Meacham et al. 2003). All the PCR fingerprinting profiles were assigned arbitrary designations, and quantitative differences among the profiles were defined using the Dice coefficient. Cluster analysis was carried out based on the unweighted pair group with arithmetic averages (UPGMA) using a position tolerance of 0.5. The single numerical index of discrimination (D) was based on the probability that two unrelated strains sampled from the test population will be placed into different typing groups. This probability can be calculated by Simpson’s index of diversity (Simpson 1972).

## Results

### Prevalence of *V. cholerae* in the fish species

In this study, a total of 3716 yellow single colonies recovered from the 40 freshwater fish samples were randomly picked out from the selective TCBS agar plates for further identification. Approximately 84.0% (3123/3716) of these colonies were tested negative in either the double-arginine hydrolase test or the esculin hydrolysis test. Moreover, they were detected positive for the *V. cholerae*-specific gene *lolB*, which is highly conserved for *V*. *cholerae* (Lalitha et al. 2008). The results were confirmed by DNA sequencing and analysis based on amplicons of the *lolB* and 16S rRNA genes. Additionally, 64.8% (2022/3123) of the *V. cholerae* isolates originated from the Jiayan aquatic market, and 35.3% (1101/3123) from the Jiangyang aquatic market. Furthermore, approximately 28.7% (*n* = 897), 20.7% (*n* = 645), 29.5% (*n* = 922), and 21.1% (*n* = 659) of the isolates were recovered from the *C*. *auratus*, *A*. *nobilis*, *P*. *pekinensis*, and *C*. *idellus* samples, respectively.

### Virulence and virulence-associated genes in the *V. cholerae* isolates

**Fig. 1:**
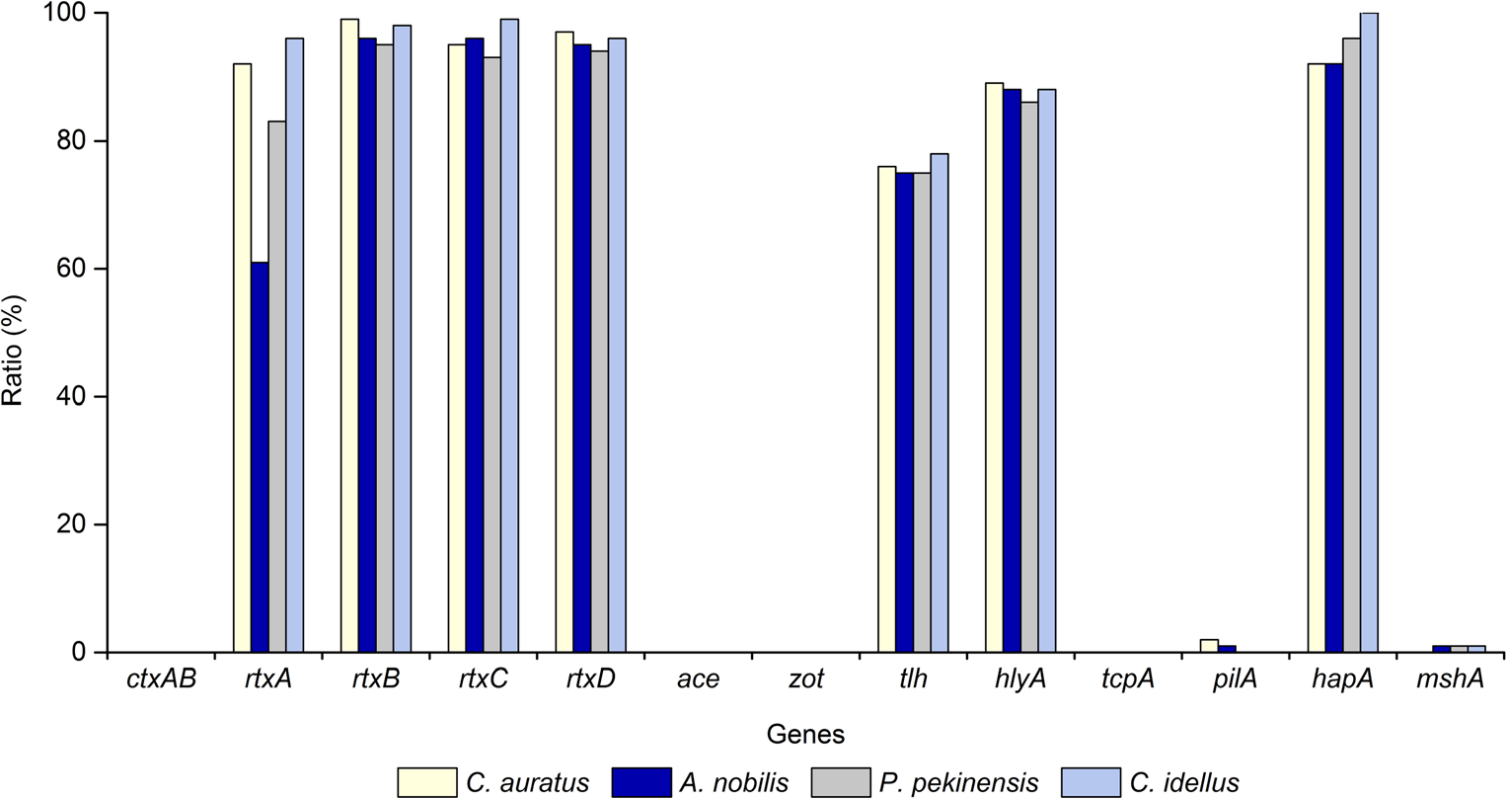
Virulence profiles of the 400 *V. cholerae* isolates recovered from the four fish species

Pure culture of randomly selected 100 *V. cholerae* isolates recovered from each species of the four commonly consumed freshwater fish was analyzed and reported in this study. All the isolates were detected positive for the *V. cholerae*-specific *lolB* gene, but negative for the toxin genes *ctxAB*, *ace*, *zot*, and *tcpA* (Fig. 1). In contrast, high occurrence of the virulence-associated genes *rtxABCD* (83.0%, 97.0%, 95.8%, and 95.5%, respectively), *hlyA* (87.8%), *tlh* (76.0%), and *hapA* (95.0%) was observed in the 400 *V. cholerae* isolates, whereas low percentages of the *pilA* (0.8%) and *mshA* (0.8%) genes were detected (Fig. 1).

As illustrated in Fig. 1, the *V. cholerae* isolates recovered from the four freshwater fish species had similar toxic genotypes; the most of which were featured with the *rtxA*, *tlh*, *hlyA*, and *hapA* genes (75.0–100.0%), except a lower percentage of *rtxA* gene in *A. nobilis* (61.0%). However, the *pilA* gene was only detected from two isolates derived from *C. auratus* and one from *A. nobilis*. The *mshA* gene was only present in three isolates, which were recovered from *A. nobilis*, *C. idellus*, and *P. pekinensis*, respectively.

### Antimicrobial resistance profiles of the *V. cholerae* isolates

**Fig. 2:**
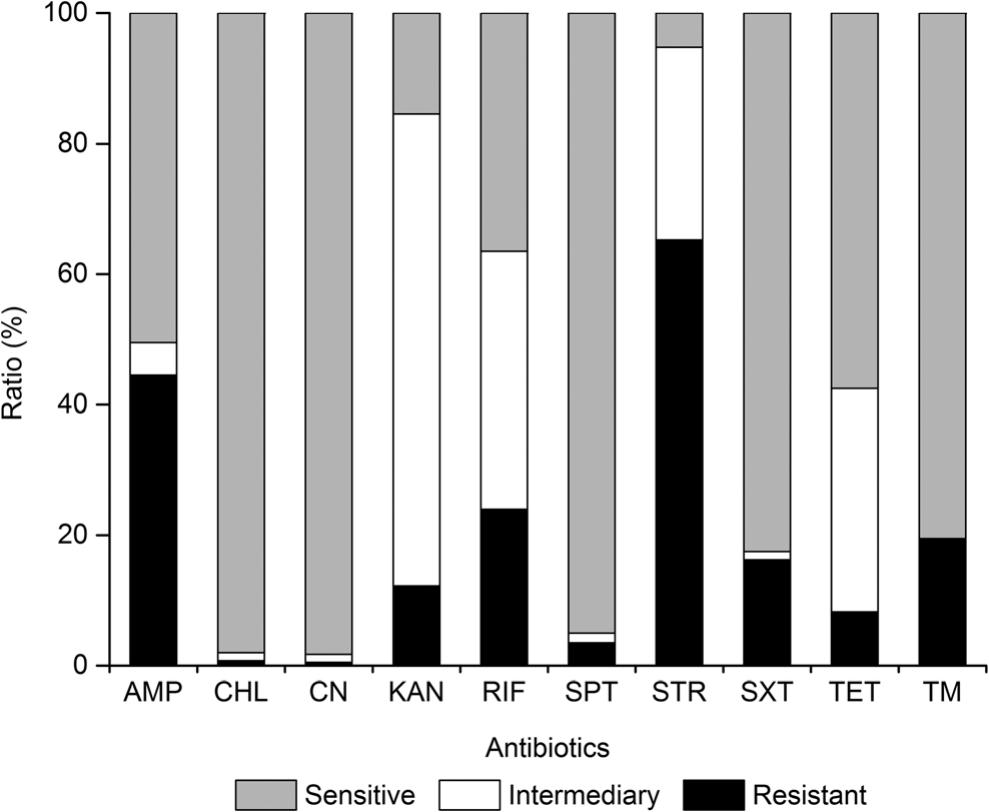
Antimicrobial susceptibility profiles of the 400 *V. cholerae* isolates evaluated in this study. *AMP* ampicillin, *CHL* chloramphenicol, *CN* gentamicinm, *KAN* kanamycin, *RIF* rifampicin, *SPT* spectinomycin, *STR* streptomycin, *SXT* sulfamethoxazole-trimethoprim, *TET* tetracycline, *TM* trimethoprim

We determined antimicrobial susceptibility in vitro of the 400 *V. cholerae* isolates to ten antimicrobial agents, and the resulting data were illustrated in Fig. 2 (Table S1). Approximately 15.3% of the isolates were susceptible to all the ten antimicrobial drugs evaluated. Moreover, most isolates were also sensitive to CN (98.3%), CHL (98.0%), and SPT (95.0%). In contrast, the STR resistance was the most predominant (65.3%) among the *V. cholerae* isolates, followed by AMP (44.5%) and RIF (24.0%). Approximately 72.3%, 39.5%, and 34.3% of the isolates also exhibited intermediate susceptibility to KAN, RIF, and TET. The resistance trend of the 400 *V. cholerae* isolates was STR > AMP > RIF > TM > SXT > KAN > TET > SPT > CHL > CN.

**Fig. 3:**
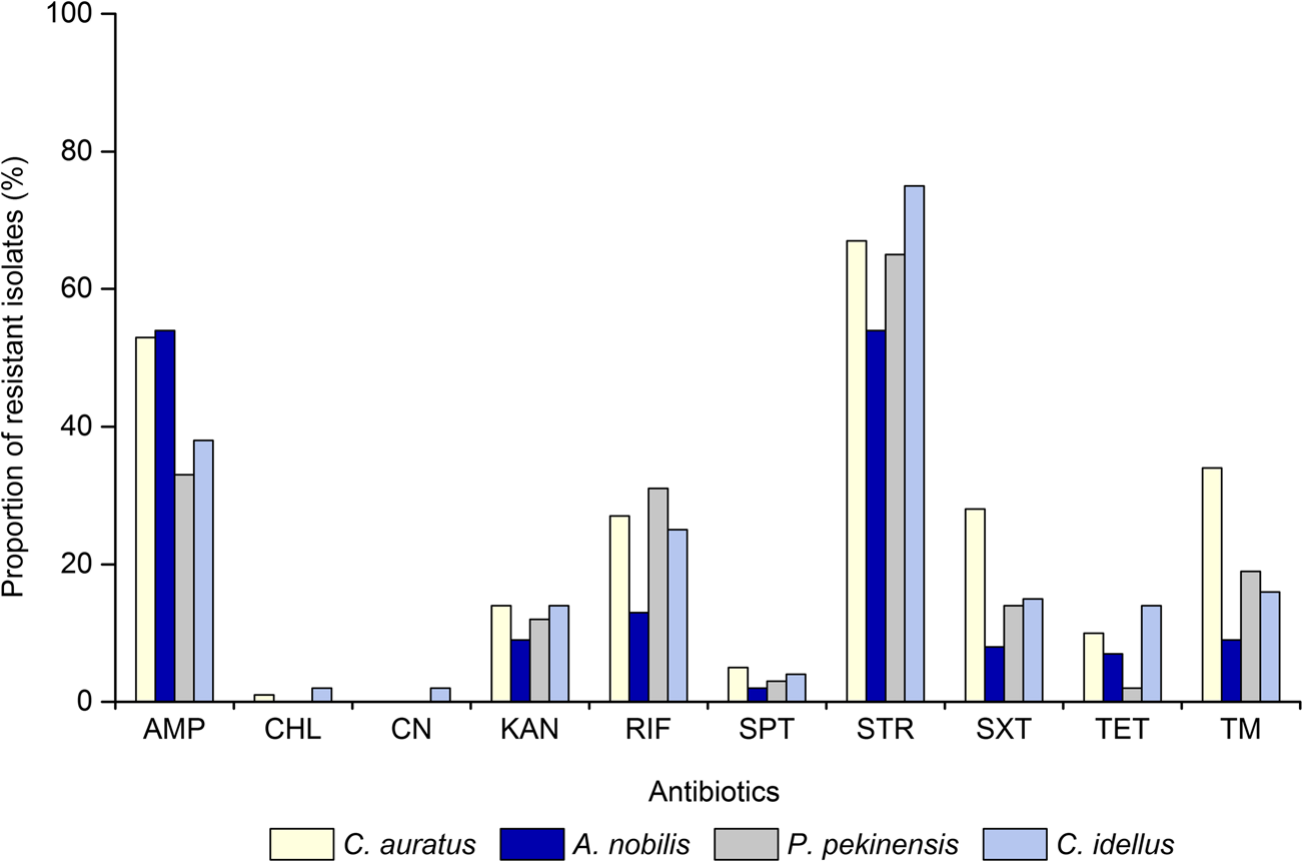
Antimicrobial resistance profiles of *V. cholerae* in the four fish species

Our data also revealed different antimicrobial-resistant profiles for the *V. cholerae* isolates recovered from different fish species (Fig. 3). The isolates from *C*. *idellus* had a higher proportion of resistance to STR (75.0%) than those isolates recovered from *C*. *auratus* (67.0%), *P*. *pekinensis* (65.0%), and *A. nobilis* (54.0%). About half of *A. nobilis* (54.0%) and *C*. *auratus* (53.0%) isolates were also resistant to AMP, which were higher than those isolates from *C*. *idellus* (38.0%) and *P*. *pekinensis* (33.0%). Meanwhile, the TM, SPT, and SXT-resistant *V. cholerae* isolates from *C*. *auratus* were 34.0%, 5.0%, and 28.0%, respectively, which were higher than those observed in *A. nobilis* (9.0%, 2.0%, and 8.0%), *P*. *pekinensis* (19.0%, 3.0%, and 14.0%), and *C*. *idellus* (16.0%, 4.0%, and 15.0%). Additionally, a few isolates from *C*. *idellus* and *C*. *auratus* were also resistant to CN and or CHL, whereas none of the isolates from the other two fish species were resistant to these two drugs.

In this study, approximately 30.5% (*n* = 122) of the isolates exhibited MDR phenotypes (Ling et al. 1983), which varied depending on the fish species. The strains isolated from *C*. *auratus* showed the highest occurrence of MDR (42.0%), followed by 34.0%, 31.0%, and 15.0% from the *C*. *idellus*, *P*. *pekinensis*, and *A. nobilis*, respectively. The values of MARI of the 400 *V. cholerae* isolates ranged from 0.00 to 0.70, indicating varying degrees of exposing to the antimicrobial agents evaluated. Forty-two different resistance patterns with a significantly different MARI (> 0.20) were observed. Two isolates recovered from *C*. *idellus* had the highest MARI of 0.70, and displayed resistance to seven of the ten antimicrobial agents tested. Additionally, the mean MARI values for the *V. cholerae* isolates recovered from *C*. *auratus*, *A*. *nobilis*, *P*. *pekinensis*, and *C*. *idellus* were 0.24, 0.16, 0.18, and 0.21, respectively, suggesting a significantly different antibiotic resistant *V. cholerae* population in the four fish species (*P* = 0.001).

### Heavy metal tolerance profiles of the *V. cholerae* isolates

Tolerance of the 400 *V. cholerae* isolates to eight heavy metals was also determined (Table 2). The maximum MICs observed in the tested isolates were 3200 μg/mL for Pb^2+^; 1600 μg/mL for Cr^3+^, Mn^2+^, and Ni^2+^; 800 μg/mL for Cd^2+^, Zn^2+^, and Hg^2+^; and 400 μg/mL for Cu^2+^, when compared with the quality control strain *E. coli* K12 (Malik and Aleem 2011). Many isolates were also tolerant to Hg^2+^ (49.3%), Zn^2+^ (30.3%), and Pb^2+^ (12.0%), and a few isolates resistant to Cd^2+^ (4.8%), Cr^3+^ (1.5%), and Ni^2+^ (0.3%). In contrast, all the isolates were non-resistant to Cu^2+^ and Mn^2+^. The tolerance trend of the 400 *V. cholerae* isolates was Hg^2+^ > Zn^2+^ > Pb^2+^ > Cd^2+^ > Cr^3+^ > Ni^2+^ > Cu^2+^ = Mn^2+^.

**Table 2:** Heavy metal tolerance of the 400 *V. cholerae* isolates evaluated in this study

Heavy metal	Number of isolates with a maximum observed MIC (μg/mL)											Resistance	
	3.125	6.25	12.5	25	50	100	200	400	800	1600	3200	No.	(%)
Cd^2+^							a						
		1	1	5	25	153	196	13	6			19	4.8
Cr^3+^									a				
								13	381	6		6	1.5
Cu^2+^								a					
					5	101	252	42				0	0.0
Hg^2+^		a											
	80	123	113	69	12		2		1			197	49.3
Mn^2+^										a			
						107	251	16	22	4		0	0.0
Ni^2+^									a				
			1		1	15	320	60	2	1		1	0.3
Pb^2+^									a				
							1	1	350	47	1	48	12.0
Zn^2+^							a						
				1		63	215	112	9			121	30.3

^a^Minimal inhibition concentration of the standard quality control strain *E. coli* K12

**Fig. 4:**
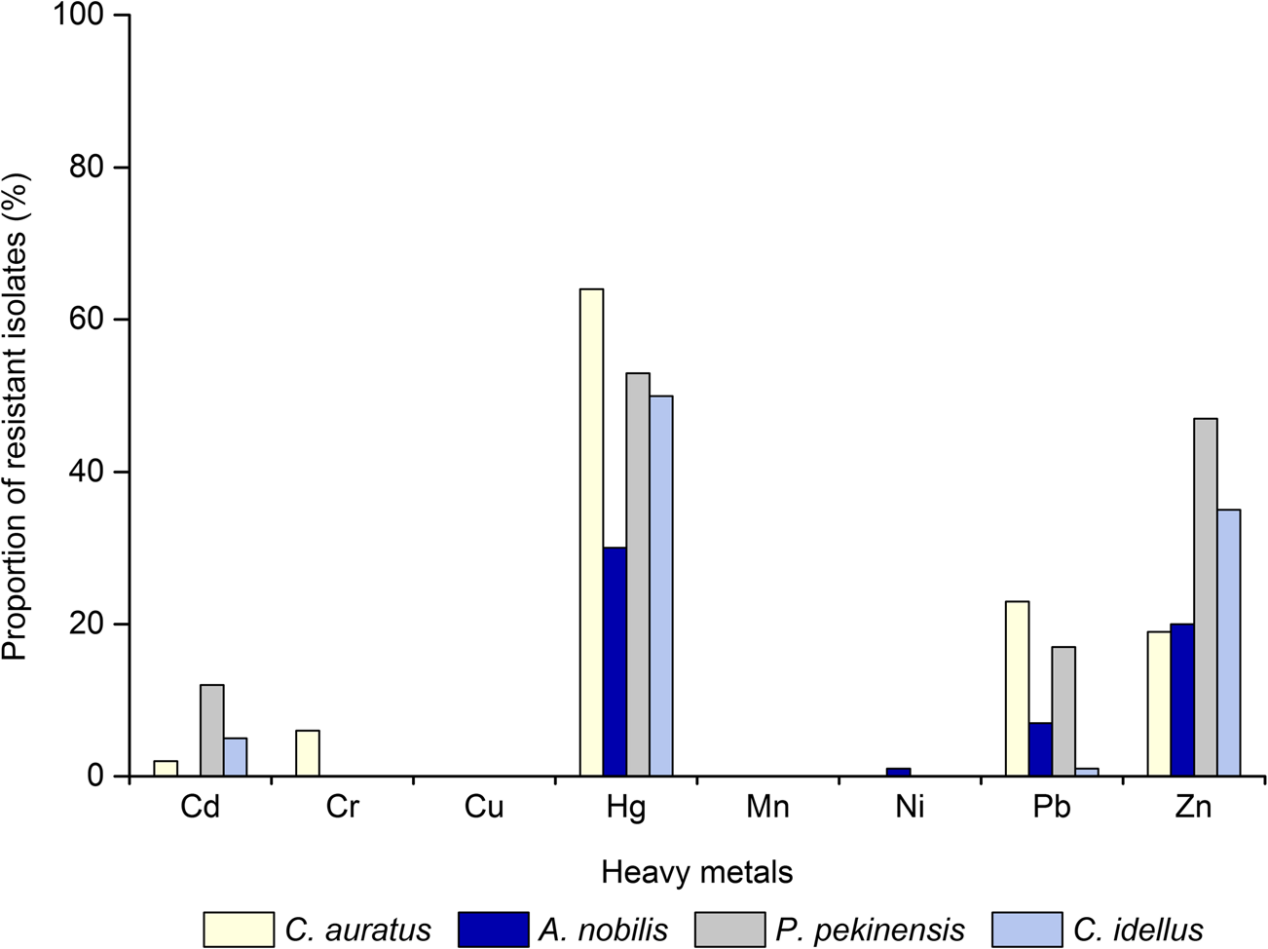
Heavy metal tolerance profiles of *V. cholerae* in the four fish species

As shown in Fig. 4, the *V. cholerae* isolates recovered from the four freshwater fish species had different heavy metal tolerance profiles. About half of the isolates from *C*. *auratus* (64.0%), *P. pekinensis* (53.0%), and *C*. *idellus* (50.0%) showed resistance to Hg^2+^, and a lower percentage from *A. nobilis* (30.0%). Approximately 47.0% and 35.0% of the isolates from *P. pekinensis* and *C. idellus* were tolerant to Zn^2+^, respectively, while 20.0% and 19.0% from *A. nobilis* and *C*. *auratus* tolerant to this heavy metal, respectively. Moreover, the isolates from *C*. *auratus* showed the highest proportion of Pb^2+^-resistance (23.0%), followed by 17.0% from *P. pekinensis*, 7.0% from *A. nobilis*, and 1.0% from *C*. *idellus*. The highest percentage of Cd^2+^-resistant isolates (12.0%) was observed from *P. pekinensis*, followed by 5.0% from *C*. *idellus* and 2.0% from *C*. *auratus*, but all of the isolates from *A. nobilis* were susceptible to this heavy metal. It is noteworthy that all of the Cr^3+^- and Ni^2+^-resistant isolates (6.0% and 1.0%) were recovered from *C*. *auratus* and *A. nobilis*, respectively.

### Genetic diversity of the *V. cholerae* isolates

The ERIC-PCR was used to investigate genetic diversity of the 400 *V. cholerae* isolates recovered from the four fish species. The obtained fingerprinting profiles comprised various numbers of DNA bands mainly ranging from 100 to 5000 bp, among which a 500-bp band was shared by all the isolates (Figure S1). Based on the fingerprinting profiles, all the isolates were classified into 328 different ERIC-genotypes, 86.9% (*n* = 285) of which were assigned as singletons. Approximately 27.0% (*n* = 77), 26.0% (*n* = 74), 24.6% (*n* = 70), and 22.4% (*n* = 64) of these singletons were derived from *A. nobilis*, *C. auratus*, *P. pekinensis*, and *C. idellus*, respectively. The UPGMA algorithm grouped all the 328 ERIC-genotypes into 12 distinct clusters at a 22.0% similarity cut-off level (Figure S1). About half (47.8%) of the 400 *V. cholerae* isolates were grouped into Cluster 5, while 20.5% (*n* = 82) were distributed into Cluster 6 (10.8%, *n* = 43) and Cluster 11 (9.8%, *n* = 39), and the remaining (31.8%, *n* = 127) were classified into Clusters 1 to 4, 7 to 10, and 12 with percentages in the range from 5.5 to 1.5% (Figure S1). Most isolates had the similarity coefficient of 30.0–90.0%, and the Simpson’s diversity index was 0.9987. These results demonstrated high genetic diversity of the 400 *V. cholerae* isolates recovered from the four fish species.

On the other hand, approximately 28.8% (*n* = 115) of the 400 *V. cholerae* isolates shared 43 ERIC-genotypes (Table S2), most of which were grouped into Cluster 5 (65.1%, *n* = 28). Among these 115 isolates, approximately 31.3% (*n* = 36) were recovered from *C. idellus*, followed by 26.1% (*n* = 30), 22.6% (*n* = 26), and 20.0% (*n* = 23) from *P. pekinensis*, *C. auratus*, and *A. nobilis*, respectively. For instance, the most predominant ERIC-genotype *vc*00124 was derived from *C. idellus* (5.2%, *n* = 6), suggesting likely near-present relatives or clonal relatedness. Likewise, the ERIC-genotypes *vc*00129 (3.5%, *n* = 4), *vc*00307 (3.5%, *n* = 4), and *vc*00148 (3.5%, *n* = 4) were derived from the *V. cholerae* isolates from *C. idellus*, *C. idellus*, and *A. nobilis*, respectively. Moreover, there were 15 genotypes (34.9%, *n* = 15) shared by the isolates derived from different fish species, suggesting possible interspecies transmission of *V. cholerae*. For instance, five isolates shared the identical genotype *vc*00067, three of which were recovered from *C. auratus* (*C. auratus*02-50, *C. Auratus* 02-65, and *C. auratus* 02-22), and two from *A. nobilis* (*A. nobilis*10-63) and *P. pekinensis* (*P. pekinensis* 08-05), respectively.

### Comparison of the MDR and heavy metal tolerance

**Fig. 5:**
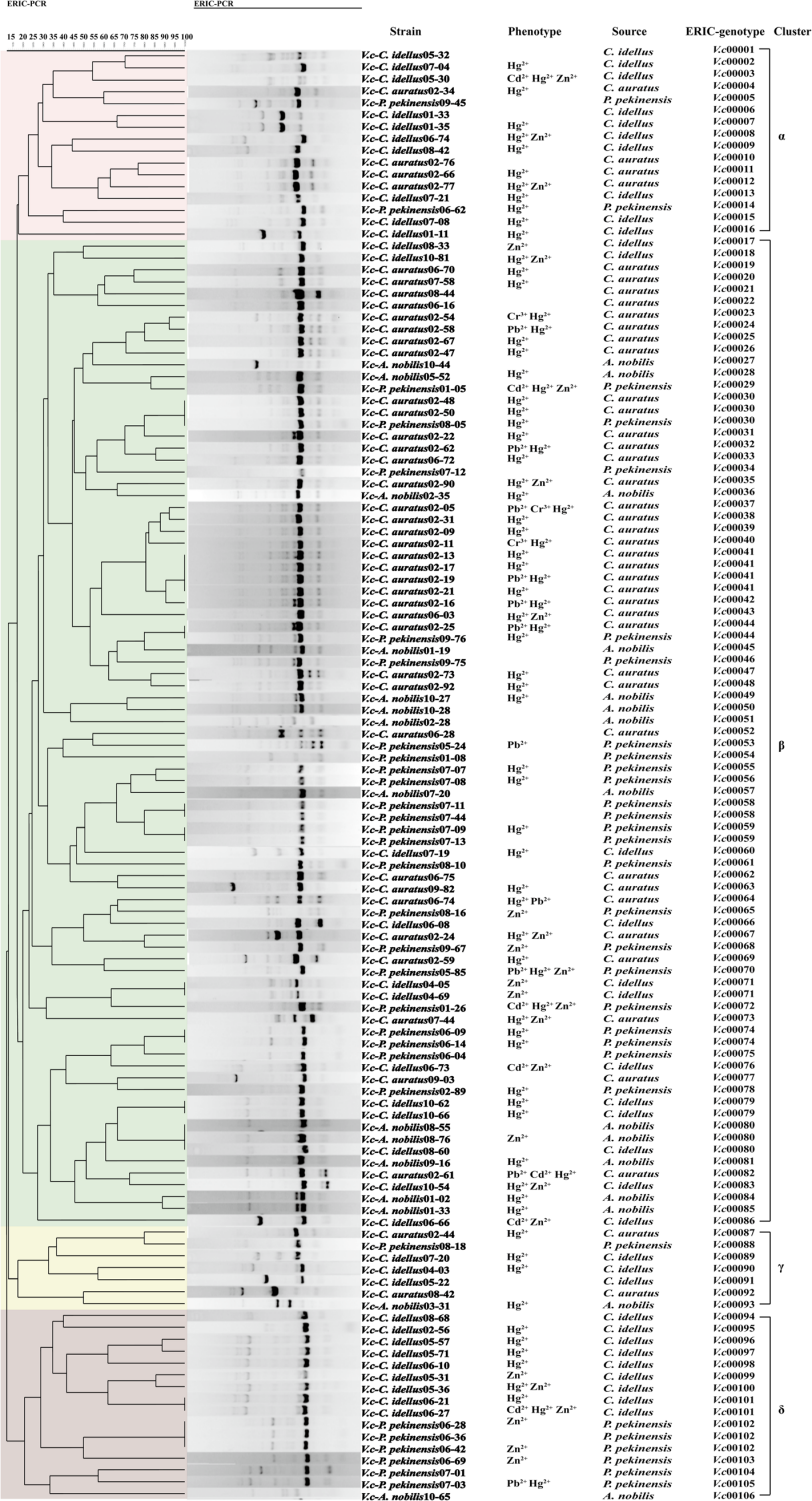
The ERIC-PCR fingerprinting profiles of the MDR *V. cholerae* isolates

The 122 *V. cholerae* isolates with MDR phenotypes were further analyzed, and the resulting data revealed the great genetic diversity with the Simpson’s diversity index of 0.9970 (Fig. 5). These MDR isolates belonging to 106 ERIC-genotypes were classified into four distinct clusters, designated as Cluster α, β, γ, and δ. The majority of the MDR isolates were grouped into Cluster β (68.0%, *n* = 83) with 70 ERIC-genotypes, about 43.4% (*n* = 36), 26.5% (*n* = 22), 15.6% (*n* = 13), and 14.5% (*n* = 12) of which were recovered from *C. auratus*, *P. pekinensis*, *A. nobilis*, and *C. idellus*, respectively. Cluster α was the second largest cluster (13.1%, 16/122) and consisted of 16 MDR isolates with 16 ERIC-genotypes, while the Cluster δ also contained 16 isolates and Cluster γ had only seven MDR isolates. Among the 122 MDR isolates, about half (51.6%, *n* = 63) were tolerant to one heavy metal, and 16.4% (*n* = 20) and 5.7% (*n* = 7) were tolerant to two and three heavy metals, respectively. Different resistance profiles were observed in different phylogenetic clusters. For instance, the Hg/AMP/RIF/STR resistance profile was the most predominant in Cluster α (25.0%, 4/16), followed by the Hg/AMP/STR/TM (18.8%, 3/16) and Hg/AMP/STR/SXT resistance profiles (12.5%, 2/16). In Cluster β, approximately 27.7% (23/83) of the isolates exhibited resistance to Hg/STR/SXT/TM, followed by Hg/AMP/RIF/STR (20.5%, 17/83) and Hg/AMP/STR/TM (18.1%, 15/83). The isolates in Cluster γ had the Hg/AMP/KAN/STR (28.6%, 2/7) and Hg/AMP/KAN/RIF resistance profiles (14.3%, 1/7), while two major resistance profiles Hg/AMP/RIF/STR (31.3%, 5/16) and Zn/AMP/SXT/TM (18.8%, 3/16) were observed in Cluster δ.

Additionally, 89.6% (*n* = 95) of the 106 ERIC-genotypes were assigned as singletons. Among these singletons, approximately 35.8% (*n* = 34), 28.4% (*n* = 27), 22.1% (*n* = 21), and 13.7% (*n* = 13) were derived from *C. auratus*, *C. idellus*, *P. pekinensis*, and *A. nobilis*, respectively. The MDR isolates with the identical ERIC-genotypes had the similar resistance profiles. For instance, two isolates (*C. auratus*02-25 and *P. pekinensis*09-76) recovered from *C. auratus* and *P. pekinensis* had the identical ERIC-genotype *vc*00036, and showed similar resistance to five antimicrobial agents (KAN/SPT/STR/SXT/TM) and one heavy metal (Hg) (Table S2).

Taken together, these data demonstrated the considerable genetic diversity of the 122 MDR *V. cholerae* isolates, as well as the close relatedness of resistance phenotypes between MDR and heavy metals.

## Discussion

*V. cholerae* is a leading waterborne pathogen worldwide. Continuous monitoring of *V. cholerae* contamination in aquatic products and identification of risk factors (e.g., virulence and transmissibility of antimicrobial resistance and heavy metal tolerance) are crucial for assuring food safety. To date, a few studies have been conducted to characterize *V. cholerae* in fish (Lan and Love 2012; Runft et al. 2014; Senderovich et al. 2010; Traore et al. 2014; Zago et al. 2017). In this study, *V. cholerae* was isolated and characterized from the four commonly consumed freshwater fish (*A*. *nobilis*, *C*. *auratus*, *C*. *idellus*, and *P*. *pekinensis*). To our knowledge, *V. cholerae* has not been previously detected for *A*. *nobilis* and *P*. *pekinensis*.

Our data revealed none occurrence of epidemic *V. cholerae* (*ctxAB*^+^*tcpA*^+^) in the fish samples evaluated in this study, consistent with some previous reports showing that neither *ctxA* nor *tcpA* is commonly expressed in environmental strains of *V. cholerae* (Traore et al. 2014; You et al. 2008). Virulence factors associated with the CTX element, such as *zot* and *ace*, were also rarely found in *V. cholerae* isolates of environmental water origin (Akoachere and Mbuntcha 2014; Bakhshi et al. 2009). For example, Li et al. isolated 16 *V. cholerae* strains from water samples collected at epidemic sites of a 2005 cholera outbreak occurred in the Nansha District of Guangzhou in China, and found that all the isolates were negative for either both *ctxA* and *tcpA* or all the four genes *ctxA*, *tcpA*, *ace*, and *zot*, except for one strain that was positive for all four genes (Li et al. 2015). Recently, Zago et al. also reported that none of 53 *V. cholerae* strains (non-O1/O139) isolated from ornamental fish species in Italy contained *ctxA*, *zot*, and *ace*. Most of the fish originated from South-East Asian countries between 2000 and 2015 (Zago et al. 2017). These reports were consistent with our findings regarding the *ace* and *zot* genes detected in the 400 *V. cholerae* isolates in this study. Previous studies have also shown that *V. cholerae* strains isolated from the environment have other virulence-associated genes such as *rtxA* and *hlyA* (Halder et al. 2017; Kumar et al. 2010). The RTX toxin gene cluster (*rtxABCD*) is essential for the cytotoxic activity of *V. cholerae* O1 El Tor strain upon Hep-2 cells in vitro test (Lin et al. 1999). The extracellular pore-forming toxin hemolysin (HlyA) can be produced by biotype El Tor of serogroup O1 and most of the non-O1/O139 strains, and has various biological activities (Benitez and Silva 2016; Gao et al. 2018). Recently, Zago et al. reported that 31.5% (*n* = 17) and 18.5% (*n* = 10) of *V. cholerae* strains isolated from ornamental fish species carried the *rtxA* and *hlyA* genes, respectively (Zago et al. 2017). In this study, our data revealed high percentages of the *rtxA* (83.0%), *rtxB* (97.0%), *rtxC* (95.8%), and *rtxD* (95.5%) genes, as well as the *hlyA* gene (87.8%) in the 400 *V. cholerae* isolates recovered from the four fish species. The *tlh* gene-encoding protein has phospholipase and lecithinase activity (Fiore et al. 1997). In this study, the *tlh* gene was detected positive in 76.0% of the 400 *V. cholerae* isolates. *V. cholerae* produces at least three morphologically distinct types of pili. Except for the TCP, the MSHA pili of *V. cholerae* is used to adhere to zooplankton exoskeletons as a survival strategy in the aquatic environment (Moorthy and Watnick 2004; Chiavelli et al. 2001). The third type of pili is encoded by a 5.4-kb *pil* gene cluster that resembles the *tap* gene cluster in *Aeromonas hydrophila* and other type IV-A pilus assembly operons in bacteria (Fullner and Mekalanos 1999). In this study, the *mshaA* and *pilA* genes were present in 0.8% and 0.8% of the 400 *V. cholerae* isolates, respectively. Additionally, the *hapA* gene encodes a hemagglutinin protease, and plays an important role in *V. cholerae* interaction with aquatic hosts (Halpern et al. 2003). Previous research has indicated that 98.0% of *V. cholerae* strains carried the *hap* gene irrespective of their source, i.e., clinical or environmental (Hasan et al. 2013). Recently, Jiang et al. reported that all three *V. cholerae* strains isolated from hepatitis B cirrhosis patients in China harbored pathogenicity-related genes *rtxA*, *rtxC*, *toxR*, *hapA*, *hlyA*, and *ompW*, but lacked *ctxA*, *ctxB*, *tcpA*, *ompU*, and *zot* genes (Jiang et al. 2018). In this study, approximately 95.0% of the 400 *V. cholerae* isolates originated from the four fish species were detected positive for the *hapA* gene. Taken together, our data in this study revealed an extremely low occurrence of pathogenic *V. cholerae* carrying the major virulence genes *ctxAB* (0.0%), *tcpA* (0.0%), *ace* (0.0%), and *zot* (0.0%), as well as potential toxin genes *mshA* (0.8%) and *pilA* (0.8%). However, high incidence of virulence-associated genes was observed, including the RTX toxin gene cluster (*rtxA*-*D*) (83.0–97.0%), *hlyA* (87.8%), *tlh* (76.0%), and *hapA* (95.0%).

Since the mid-1980s, MDR has considerably increased in toxicogenic *V. cholerae* (Ghosh and Ramamurthy 2011; Kitaoka et al. 2011). In this study, approximately 30.5% of the 400 *V. cholerae* isolates recovered from the four fish species had MDR phenotypes, and two isolates derived from *C. idellus* were resistant to the highest number of antimicrobial agents (7 of 10). The MARI is often used to determine the antibiotic resistance-associated health risk (Fri et al. 2018). In this study, the identified 42 resistance patterns had a significantly different MARI (> 0.20), indicating different antimicrobial exposure levels or contaminated source. Furthermore, the significant difference in antimicrobial resistance profiles among the four fish samples was also observed (MARI, *P* = 0.001), suggesting that *C*. *auratus* was likely exposed to antimicrobial drugs mostly. Additionally, STR (65.3%) resistance was very prevalent among the *V. cholerae* isolates, many of which were also resistant to AMP (44.5%) or RIF (24.0%). Baron et al. also reported high levels of resistance to STR (22.2%) and AMP (9.1%) among 99 *V*. *cholerae* strains (non-O1/O139) isolated from wastewater and shellfish in 2000/2001 in the La Rance estuary (Brittany, France) (Baron et al. 2017). High percentages of resistance to STR were also detected among toxigenic (97.9%) and non-toxigenic O139 strains (88.0%) isolated in China from 1993 to 2009 (Yu et al. 2012). In fact, antimicrobial agents such as STR, AMP, and sulfisoxazone have been frequently used in agriculture for the last some decades, and STR was the commonly used antibiotic to prevent fish diseases (Yilmaz and Sova 2018). Therefore, the high proportion of resistance of *V. cholerae* to STR could be facilitated by the selective pressure. Previous studies have also revealed high percentages of AMP-resistant *V. cholerae*. For example, Thapa Shrestha et al. reported that all the 24 *V. cholerae* isolates derived from the clinical and water samples were resistant to AMP (Thapa Shrestha et al. 2015). Recently, Ahmed et al. also reported that the AMP-resistant percentage of *V. cholerae* strains in clinical and aquatic *Vibrio* spp. isolates was 100.0%, suggesting intrinsic resistance of *Vibrio* spp. to AMP (Ahmed et al. 2018). The AMP-resistant bacteria may be attributed to the abuse of drugs and the inappropriate release of industrial wastes into the environment (Taviani et al. 2008). In this study, the low occurrence of the resistance to CN (0.5%) and CHL (0.8%) was observed in the *V. cholerae* isolates. It has also been reported that none of the 42 *V. cholerae* isolates recovered from shrimp collected in 2013 and 2014 in Shanghai, China, were resistant to CHL (He et al. 2015). The low percentage of CHL-resistant isolates may be explained by the drug and its salts and esters (including chloramphenicol succinate) have been banned from the animal breeding industry in China (China Department of Agriculture, Bulletin No.193). TET, sulfonamides, and quinolones are widely used in aquaculture. In this study, the resistance to TET was detected positive in 8.3% of the 400 *V. cholerae* isolates. It has been reported that about 11% of 550 *V. cholerae* O1 El Tor strains isolated from the seventh cholera pandemic in China from 1961 to 2010 were resistant to TET (Wang et al. 2012). Recently, Ottaviani et al. also reported a lower incidence of the TET resistance in *V. cholerae* strains (3 of 42) isolated from the sea food, sea water, and fresh water in Italy (Ottaviani et al. 2018). Additionally, in this study, the high percentages of intermediate susceptibility to KAN (72.3%), RIF (39.5%), and TET (34.3%) may suggest a potential resistance trend for these drugs.

The rapid development of industrialization, urbanization, and agricultural modernization during recent decades has resulted in the increasing pollution, such as heavy metals, in both freshwater and marine environment (Devlin 2006; Sun et al. 2018). Heavy metals have been detected from sediments of the fish farming environment (He et al. 2012), which could finally be taken into humans through food chain (Ramirez-Perez et al. 2004; Sawasdee and Kohler 2010). High occurrence of resistance to heavy metals has been reported in *Pseudomonas* spp. isolated from the environment (e.g., marine, river, and agricultural soil) (Malik and Aleem 2011). Nevertheless, very few studies have been conducted to address heavy metal tolerance of *V. cholerae* originated from the CNCO particularly from fish. In this study, for the first time, our data revealed that 49.3%, 30.3%, and 12.0% of the 400 *V. cholerae* isolates recovered from the four commonly consumed freshwater fish species were tolerant to Hg^2+^, Zn^2+^, and Pb^2+^, respectively. Xing et al. also reported that in the turbot gastrointestinal metagenome, cobalt-zinc-cadmium resistances were one of the largest categories of resistance to the toxic compound subsystem (Xing et al. 2013). In this study, about 24.0% of the 400 *V. cholerae* isolates were resistant to two or more heavy metals, and one *V. cholerae* isolate derived from *C*. *auratus* was resistant to the highest number of heavy metals (4 of 8). These data suggested heavy metal contamination in the aquacultural environment, which may originate from industrial pollutions, runoffs from farmlands, or hospital waste that finally ends up in rivers and estuaries. Furthermore, the *V. cholerae* isolates from the four fish species had different heavy metal tolerance patterns, suggesting varying degrees of the exposure to the heavy metals evaluated.

The ERIC-PCR has widely been applied to analyze bacterial genotyping in epidemiology (Ranjbar et al. 2010). It has also been used for the analysis of clonal diversity and genotypic variability of *V. cholera* strains from aquatic environment (Goel and Jiang 2011; Rivera et al. 1995). Previous studies reported that ERIC-PCR yielded one to eight DNA amplicons in the size ranging from 100 to 4000 bp (Rivera et al. 1995; Colombo et al. 1997). In this study, the established ERIC-PCR condition generated the amplicons mainly ranged from 100 to 5000 bp in size. Moreover, in this study, the 400 *V. cholerae* isolates were differentiated into 328 ERIC-genotypes (82.0%), which was more than 179 pulsotypes (71.6%) from 250 *V. cholerae* isolates, and 218 pulsotypes (38.4%) from 568 *V. cholerae* isolates based on *Not*I-PFGE (pulsed-field gel electrophoresis) genotyping technique (Gu et al. 2014; Lu et al. 2017). These data demonstrated the considerable genetic diversity of the 400 *V. cholerae* isolates recovered from the four freshwater fish species.

Previous research highlighted that novel selective pressure from the discharge of heavy metals could have significant impact on the environmental selection of antibiotic resistance genes (Alonso et al. 2001). In this study, comparison of the fingerprinting profiles derived from the MDR *V. cholerae* isolates revealed a close relatedness between the MDR and heavy metal resistance phenotypes, suggesting that heavy metal pollution most likely selects for antibiotic resistance and vice versa (Baker-Austin et al. 2006). The DNA transfer between antimicrobial-resistant bacteria from fish hatcheries and other pathogenic bacteria has been reported (Rhodes et al. 2000), which imposes potential threat upon human health. Thus, in the future studies, continuous monitoring of genetic evolution of *V. cholerae* in fish considered as natural reservoirs and vectors of resistance (Laviad-Shitrit et al. 2018) and identification of risk factors are crucial for assuring food safety and human health.

## Conclusions

In this study, for the first time, we isolated and characterized *V. cholerae* from *A*. *nobilis* and *P*. *pekinensis*, and determined heavy metal tolerance profiles of 400 *V. cholerae* isolates recovered from four commonly consumed freshwater fish (*A*. *nobilis*, *C*. *auratus*, *C*. *idellus*, and *P*. *pekinensis*) collected in July and August of 2017 in Shanghai, China. Also, our data revealed an extremely low occurrence of pathogenic *V. cholerae* carrying the major virulence genes *ctxAB* (0.0%), *tcpA* (0.0%), *ace* (0.0%), and *zot* (0.0%), as well as potential toxin genes *mshA* (0.8%) and *pilA* (0.8%). However, high incidence of virulence-associated genes was observed, including the RTX toxin gene cluster (*rtxA*-*D*) (83.0–97.0%), *hlyA* (87.8%), *tlh* (76.0%), and *hapA* (95.0%). Meanwhile, high percentages of resistance to antimicrobial agents STR (65.3%), AMP (44.5%), and RIF (24.0%) were also observed. Approximately 30.5% (122/400) of the isolates displayed MDR phenotypes with 42 different resistance patterns (MARI > 0.20), which were significantly different among the four fish species (MARI, *P* = 0.001). Additionally, tolerance of isolates to heavy metals Hg^2+^ (49.3%), Zn^2+^ (30.3%), and Pb^2+^ (12.0%) was observed. Approximately 73.8% (90/122) of the MDR isolates were also tolerant to heavy metals evaluated. *C*. *auratus* was likely exposed to antimicrobial drugs and heavy metals mostly when compared with the other fish species. The ERIC-PCR-based fingerprinting of the 400 *V. cholerae* isolates revealed 328 ERIC-genotypes, and the MDR isolates were classified into 106 ERIC-genotypes, which demonstrated a large degree of genomic variation among the isolates. Overall, the results in this study revealed divergent virulence, resistance phenotypes, and ERIC-genotypes of the *V. cholerae* isolates recovered from the four fish species. The increasing resistance of *V. cholerae* imposed potential threat upon human health. Therefore, governments particularly in developing nations, aquaculture industry, and food consumers should work together toward eliminating and controlling the leading waterborne pathogen worldwide. In the future research, molecular mechanisms underlying the co-selection for antimicrobial and heavy metal resistant *V. cholerae* will be investigated.

## Electronic supplementary material

Table S1 Antimicrobial susceptibility rates of the 400 *V. cholerae* isolates evaluated in this study (DOC 32 kb)

Table S2 The resistance profiles of the *V. cholerae* isolates with similar ERIC-genotypes (DOC 151 kb)

Fig. S1 The ERIC-PCR fingerprinting profiles of 400 *V. cholerae* isolates evaluated in this study (PNG 19357 kb)

High resolution image (TIF 54576 kb)
