# Supplementary material for: Virulence, antimicrobial and heavy metal tolerance, and genetic diversity of Vibrio cholerae recovered from commonly consumed freshwater fish
Source: Environ Sci Pollut Res Int. 2019 Jul 19;26(26):27338–52. doi: 10.1007/s11356-019-05287-8 (PMC6733808; doi:10.1007/s11356-019-05287-8)
Supplement: Supplementary file 1 — Antimicrobial susceptibility rates of the 400 V. cholerae isolates evaluated in this study (DOC 32 kb) [file 11356_2019_5287_MOESM1_ESM.doc]

**Table S1** **Antimicrobial susceptibility rates of the 400 *V. cholerae* isolates evaluated in this study**

| Antimicrobial agent | Antimicrobial susceptibility rate (%) | | |
| --- | --- | --- | --- |
|  | Resistant | Intermediary | Sensitive |
| AMP | 44.5 | 5 | 50.5 |
| CHL | 0.8 | 1.3 | 98 |
| CN | 0.5 | 1.3 | 98.3 |
| KAN | 12.3 | 72.3 | 15.5 |
| RIF | 24 | 39.5 | 36.5 |
| SPT | 3.5 | 1.5 | 95 |
| STR | 65.3 | 29.5 | 5.3 |
| SXT | 16.3 | 1.3 | 82.5 |
| TET | 8.3 | 34.3 | 57.5 |
| TM | 19.5 | 0 | 80.5 |
