# Supplementary material for: Virulence, antimicrobial and heavy metal tolerance, and genetic diversity of Vibrio cholerae recovered from commonly consumed freshwater fish
Source: Environ Sci Pollut Res Int. 2019 Jul 19;26(26):27338–52. doi: 10.1007/s11356-019-05287-8 (PMC6733808; doi:10.1007/s11356-019-05287-8)
Supplement: Supplementary file 2 — The resistance profiles of the V. cholerae isolates with similar ERIC-genotypes (DOC 151 kb) [file 11356_2019_5287_MOESM2_ESM.doc]

**Table S2** The resistance profiles of the *V. cholerae* isolates with similar ERIC-genotypes

| Cluster | ERIC-genotype | Strain | Antimicrobial resistance profile | Heavy metal resistance profile |
| --- | --- | --- | --- | --- |
| 1 | *vc*00005 | *C. idellus*04-03 | AMP/KAN/STR | Hg^2+^ |
|  |  | *C. idellus*04-41 | AMP/STR | Hg^2+^ |
| 3 | *vc*00016 | *C. idellus*08-33 | RIF/STR | Zn^2+^ |
|  |  | *C. idellus*08-54 | STR | Hg^2+^ |
| 4 | *vc*00021 | *A. nobilis*08-55 | AMP/KAN/STR | -^*^ |
|  |  | *A. nobilis*08-76 | AMP/RIF/STR/TET | Zn^2+^ |
|  |  | *C. idellus*08-60 | SPT/STR/SXT/TM | - |
| 5 | *vc*00036 | *C. auratus*02-25 | KAN/ SPT/STR/SXT/TM | Hg^2+^/Pb^2+^ |
|  |  | *P. pekinensis*09-76 | KAN/RIF/SPT/STR/SXT/TM | Hg^2+^ |
|  | *vc*00057 | *C. auratus*02-13 | AMP/RIF/STR/SXT/TM | Hg^2+^ |
|  |  | *C. auratus*02-17 | AMP/STR/SXT/TET/TM | Hg^2+^ |
|  |  | *C. auratus*02-19 | AMP/STR/SXT/TM | Hg^2+^/Pb^2+^ |
|  |  | *C. auratus*02-21 | AMP/STR/SXT/TM | Hg^2+^ |
|  | *vc*00059 | *C. auratus*02-11 | AMP/RIF/STR/SXT/TM | Cr^3+^/ Hg^2+^ |
|  |  | *C. auratus*02-23 | AMP/TM | Hg^2+^ |
|  |  | *C. auratus*02-31 | AMP/RIF/STR/SXT/TM | Hg^2+^ |
|  | *vc*00063 | *P. pekinensis*06-64 | - | Hg^2+^/Pb^2+^/Zn^2+^ |
|  |  | *P. pekinensis*09-43 | RIF/STR | Pb^2+^/Zn^2+^ |
|  | *vc*00067 | *C. auratus*02-50 | AMP/KAN/SPT/STR/SXT/TM | Hg^2+^ |
|  |  | *C. auratus*02-65 | RIF | Zn^2+^ |
|  |  | *A. nobilis*10-63 | AMP/STR | Pb^2+^ |
|  |  | *P. pekinensis*08-05 | AMP/KAN/RIF/STR | Hg^2+^ |
|  |  | *C. auratus*02-22 | AMP/STR/SXT/TM | Hg^2+^ |
|  | *vc*00070 | *C. auratus*09-88 | RIF | - |
|  |  | *C. auratus*02-62 | AMP/RIF/SPT/SXT/TM | Hg^2+^/Pb^2+^ |
|  | *vc*00076 | *C. auratus*02-79 | AMP/STR | Hg^2+^/Pb^2+^ |
|  |  | *C. auratus*02-45 | AMP/STR | Hg^2+^ |
|  | *vc*00107 | *P. pekinensis*06-14 | AMP/SXT/TM | Hg^2+^ |
|  |  | *P. pekinensis*06-04 | AMP/SXT/TM | - |
|  | *vc*00110 | *C. idellus*01-20 | STR | Hg^2+^ |
|  |  | *C. idellus*01-39 | - | Hg^2+^/Zn^2+^ |
|  |  | *C. idellus*01-40 | - | Hg^2+^/Zn^2+^ |
|  |  | *A. nobilis*09-12 | RIF | - |
|  | *vc*00120 | *C. auratus*09-17 | RIF/STR | Pb^2+^ |
|  |  | *C. auratus*09-05 | STR | Hg^2+^ |
|  | *vc*00121 | *P. pekinensis*05-04 | - | Hg^2+^/Pb^2+^ |
|  |  | *P. pekinensis*05-82 | - | Cd^2+^/Hg^2+^/Zn^2+^ |
|  |  | *P. pekinensis*05-86 | STR | Hg^2+^/Pb^2+^/Zn^2+^ |
|  |  | *C. idellus*10-77 | STR | Zn^2+^ |
|  | *vc*00122 | *C. idellus*02-25 | RIF/STR | - |
|  |  | *P. pekinensis*02-57 | STR | Zn^2+^ |
|  | *vc*00124 | *C. idellus*10-82 | AMP/STR | Hg^2+^ |
|  |  | *C. idellus*10-61 | AMP/STR | Hg^2+^ |
|  |  | *C. idellus*10-62 | AMP/KAN/STR | Hg^2+^ |
|  |  | *C. idellus*10-66 | AMP/KAN/RIF/STR | Hg^2+^ |
|  |  | *C. idellus*10-79 | AMP/STR | Hg^2+^ |
|  |  | *C. idellus*10-80 | AMP/STR | Hg^2+^ |
|  | *vc*00126 | *P. pekinensis*02-63 | STR | Cd^2+^/Hg^2+^ |
|  |  | *P. pekinensis*02-89 | KAN/SPT/STR/SXT/TM | Hg^2+^ |
|  |  | *C. idellus*02-42 | - | - |
|  |  | *C. auratus*06-16 | KAN/STR/SXT/TET/TM | - |
|  | *vc*00129 | *C. idellus*04-20 | STR | - |
|  |  | *C. idellus*04-28 | RIF/STR | Zn^2+^ |
|  |  | *C. idellus*05-22 | AMP/KAN/RIF/STR | - |
|  |  | *C. idellus*02-31 | - | Zn^2+^ |
|  | *vc*00131 | *A. nobilis*01-33 | AMP/RIF/STR | Hg^2+^ |
|  |  | *A. nobilis*01-01 | STR | - |
|  | *vc*00132 | *A. nobilis*01-02 | AMP/KAN/STR | Hg^2+^ |
|  |  | *A. nobilis*10-58 | AMP | - |
|  | *vc*00133 | *A. nobilis*10-59 | AMP | - |
|  |  | *A. nobilis*01-46 | AMP/STR | Hg^2+^/Zn^2+^ |
|  | *vc*00140 | *A. nobilis*10-12 | AMP | - |
|  |  | *A. nobilis*10-16 | AMP/STR | Pb^2+^ |
|  |  | *A. nobilis*10-15 | AMP/STR | - |
|  | *vc*00148 | *A. nobilis*03-04 | AMP/STR | - |
|  |  | *A. nobilis*03-59 | AMP | - |
|  |  | *A. nobilis*03-61 | AMP/STR | - |
|  |  | *A. nobilis*10-48 | AMP/STR | - |
|  | *vc*00149 | *A. nobilis*03-10 | AMP/STR | Hg^2+^ |
|  |  | *A. nobilis*10-51 | AMP | - |
|  | *vc*00155 | *P. pekinensis*07-44 | AMP/RIF/STR | - |
|  |  | *P. pekinensis*07-09 | AMP/RIF/STR | Hg^2+^ |
|  | *vc*00156 | *P. pekinensis*07-13 | AMP/RIF/STR | - |
|  |  | *P. pekinensis*08-02 | AMP/STR | Hg^2+^ |
|  |  | *C. auratus*02-53 | AMP/STR | Hg^2+^ |
|  | *vc*00161 | *P. pekinensis*07-12 | AMP/RIF/STR | - |
|  |  | *C. auratus*09-63 | AMP/STR | Cr^3+^/Hg^2+^/Pb^2+^/Zn^2+^ |
|  | *vc*00163 | *C. auratus*09-60 | STR | Hg^2+^/Zn^2+^ |
|  |  | *P. pekinensis*08-01 | AMP | Hg^2+^ |
|  |  | *C. auratus*08-40 | STR | Pb^2+^ |
|  | *vc*00164 | *P. pekinensis*08-03 | STR | Zn^2+^ |
|  |  | *A. nobilis*09-14 | STR/TET | Zn^2+^ |
|  | *vc*00169 | *C. auratus*02-32 | AMP/STR | Hg^2+^ |
|  |  | *C. auratus*02-01 | AMP/STR | Hg^2+^ |
|  | *vc*00173 | *C. idellus*01-18 | STR | Hg^2+^ |
|  |  | *C. idellus*01-01 | - | Hg^2+^/Zn^2+^ |
| 8 | *vc*00234 | *C. idellus*04-69 | STR/SXT/TM | Zn^2+^ |
|  |  | *C. idellus*04-75 | SXT/TM | - |
|  |  | *C. idellus*04-43 | SXT/TM | Zn^2+^ |
| 9 | *vc*00253 | *C. idellus*02-58 | STR | - |
|  |  | *C. idellus*02-64 | STR | Zn^2+^ |
|  |  | *C. idellus*02-70 | STR | - |
|  | *vc*00265 | *P. pekinensis*08-13 | AMP | Zn^2+^ |
|  |  | *C. auratus*10-43 | RIF | Zn^2+^ |
|  | *vc*00266 | *A. nobilis*02-35 | KAN/STR/SXT/TM | Hg^2+^ |
|  |  | *P. pekinensis*08-08 | AMP/STR | Hg^2+^/Pb^2+^/Zn^2+^ |
| 10 | *vc*00271 | *P. pekinensis*08-21 | STR | - |
|  |  | *P. pekinensis*08-19 | - | Zn^2+^ |
|  | *vc*00272 | *P. pekinensis*08-23 | STR | Zn^2+^ |
|  |  | *P. pekinensis*08-24 | - | Hg^2+^ |
|  | *vc*00274 | *A. nobilis*02-20 | AMP | Hg^2+^ |
|  |  | *A. nobilis*05-02 | AMP | - |
|  | *vc*00281 | *C. auratus*02-44 | AMP/RIF/SXT/TM | Hg^2+^ |
|  |  | *P. pekinensis*08-18 | AMP/KAN/RIF/STR | - |
| 11 | *vc*00290 | *C. idellus*05-91 | AMP/STR | Hg^2+^ |
|  |  | *C. idellus*05-57 | AMP/RIF/STR | Hg^2+^ |
|  | *vc*00297 | *P. pekinensis*06-24 | - | Cd^2+^/Hg^2+^ |
|  |  | *C. idellus*02-56 | AMP/SXT/TM | Hg^2+^ |
|  | *vc*00302 | *P. pekinensis*06-36 | AMP/SXT/TM | - |
|  |  | *P. pekinensis*06-42 | AMP/SXT/TM | Zn^2+^ |
|  |  | *P. pekinensis*06-58 | STR | Hg^2+^ |
|  | *vc*00307 | *C. idellus*06-21 | AMP/KAN/RIF/STR | Hg^2+^ |
|  |  | *C. idellus*06-22 | AMP/STR | Hg^2+^ |
|  |  | *C. idellus*06-23 | AMP/STR | - |
|  |  | *C. idellus*06-27 | AMP/CN/STR/TET | Cd^2+^/Hg^2+^/Zn^2+^ |

^*^ not detected.
